# Macroinvertebrate assemblages from a stream-wetland complex: a case study with implications for assessing restored hydrologic functions

**DOI:** 10.1007/s10661-023-10983-7

**Published:** 2023-02-13

**Authors:** Amy Braccia, Jamie Lau, Jesse Robinson, Michael Croasdaile, Jeong Park, Art Parola

**Affiliations:** 1 Department of Biological Sciences, Eastern Kentucky University, Richmond, KY 40475 USA; 2 Department of Biology, Radford University, Radford, VA 24142 USA; 3 J.B. Speed School of Engineering, Stream Institute, University of Louisville, Louisville, KY 40292 USA

**Keywords:** Stage 0 stream restoration, Hydrologic functions, Biological indicators, Post-restoration monitoring

## Abstract

Legacies of past land use persist today in the form of incised, single-threaded stream channels with dramatically different hydrologic functions of pre-colonial stream valleys. Restoration practices that aim to return lost hydrologic functions by re-establishing floodplain and groundwater connections should result in stream habitat and biological assemblages that differ from modern, single-threaded channels. The aim of this case study was to identify attributes of macroinvertebrate assemblages that might serve as biological indicators of improved hydrologic functions following the restoration of a stream-wetland complex, similar to a Stage 0 restoration, of a headwater valley in the Western Allegheny region of the USA. We monitored hydrologic functions and macroinvertebrate assemblages from stream reaches of a restored and unrestored site over multiple years during the early years following restoration. Reduced bed mobility and increased flow duration indicated improved hydrologic functions from the restored site. Aggregate metrics that capture functional attributes of macroinvertebrate assemblage (i.e., density and biomass) were consistently greater from the restored site. EPT biomass from restored pools was 3–4 × greater than amounts from the unrestored site as a result of consistently greater mayfly abundance. Restored pools also supported a subassemblage of taxa with life history attributes that are aligned with habitat conditions created from improved hydrologic functions. Results from this case study demonstrate the importance of habitat-specific sampling designs that report the absolute abundance of potential biological indicators. Findings from this case study should help guide the development of rapid biological indicators of improved hydrologic functions.

**Supplementary Information:**

The online version contains supplementary material available at 10.1007/s10661-023-10983-7.

## Introduction

The morphology of headwater streams influences the transport of water and sediment, and consequently, the morphology and habitat for biota are the product of those same fluxes. Prior to the European settlement of North America, this complex interaction was largely influenced by beaver. Beaver-induced wood inputs maintained floodplain connections that promoted sediment and organic matter retention and resulted in a pre-colonial landscape dominated by beaver pools and connected wetlands (Elliott et al., 2013; Naiman et al., 1988). Extirpation of beaver through the early 1900s, coupled with high-impact floodplain development (i.e., post-settlement land clearing and agriculture), wood removal, and channelization, disrupted these hydrologic connections and dramatically altered stream corridor structure and function (Foster et al., 2003; Wohl, 2004). Legacies of these past human impacts persist today in the form of single-threaded channels with a reduced capacity to attenuate floods and retain sediments and nutrients (Walter & Merritts, 2008; Wohl & Beckman, 2014).

The hydrologic functions and physical habitat of modern single-thread streams are functionally very different from pre-settlement conditions. By recognizing this difference, practitioners have developed new methods of restoring stream channels that better represent the historical baseline condition. These pre-settlement target conditions have been variously termed “stream-wetland complexes” or “stage 0 channels” (Cluer & Thorne, 2014; Kaushal et al., 2014), are typically anabranched, have frequently inundated floodplains, and have diverse but stable in-channel habitats. Stream-wetland complexes have been documented in many different environments (e.g., Collins et al., 2003; Harwood & Brown, 1993) and have served as the objective for stream restorations across the USA, including in the Pacific Northwest (e.g., Flitcroft et al., 2022), the Northeast (e.g., Goerman et al., 2013; Kaushal et al., 2014), and the Southeast (e.g., Parola & Hansen, 2011). A common goal for these restoration projects was to re-establish the connection between the stream and its floodplain, frequently leading to the formation of streamside wetlands. Some restoration projects make the reconnection of the stream to the valley groundwater an explicit goal (Parola & Hansen, 2011). In many cases, beavers have recolonized sites post restoration, and the restoration is expected to undergo successional changes associated with beaver-controlled systems (as described in Burchsted et al., 2010).

Restoring hydrologic connections should result in flow regimes and channel conditions that provide habitat for improved ecosystem functions. Post-restoration studies that simultaneously monitor restored hydrologic functions and biological assemblages that drive ecosystem processes (i.e., nutrient cycling, decomposition) are greatly needed to guide ecologically sustainable restoration practices (Harman et al., 2012; Kollmann et al., 2016). Benthic macroinvertebrates — more specifically the aquatic insects — are a diverse group of animals that play critical roles in ecosystem functions (e.g., organic matter processing, nutrient cycling, transfer of biomass and energy to adjacent terrestrial ecosystems) (Huryn & Wallace, 2000; Wallace & Webster, 1996). Structural attributes of macroinvertebrate assemblages frequently reflect environmental change because the constituent taxa represent a diversity of life history, morphological features, and behavioral adaptations for life in freshwater habitats. Thus, they are the most widely used organisms in water quality monitoring programs (Buss et al., 2015; Resh & McElravy, 1993). Given their importance to ecosystem functions and successful use as bioindicators for water quality monitoring, macroinvertebrate assemblages have the potential to serve as indicators of the ecological lift resulting from restored hydrologic functions in stream-wetland complexes.

We stress that stream-wetland complex restorations do not aim to restore aquatic assemblages to what is termed *least impacted* or *best attainable* reference conditions (sensu Stoddard et al., 2006**)** for water quality assessment and monitoring; rather, they return the hydrologic functions upon which stream ecosystem structure and function depend. While valley floors buried by legacy sediments, subfossils (Elliott et al., 2013), and ancient beaver dams (Kramer et al., 2012) can provide clues to guide restoration design, there are no *reference targets* (sensu Chapman, 1998) or regional benchmarks for the biological structure and functions of pre-colonial stream-wetland complexes. Reference targets for many stream restoration are currently selected from the best attainable conditions, but these least-disturbed conditions are morphologically and ecologically dissimilar to pre-colonial stream-wetland complexes (Burchsted et al., 2010). Developing reference targets for restored stream-wetland complexes requires quantifying the biological structure and function during the successional stages of restored valleys.

Physical and biological data from the early post-restoration years following the restoration of stream-wetland complexes is limited and is just now beginning to appear in the published literature (Flitcroft et al., 2022). The objective of our study was to compare benthic macroinvertebrate assemblages from a recently restored stream-wetland complex to an unrestored, single-threaded channel with impaired hydrologic functions. We monitored hydrologic functions and the macroinvertebrate assemblages from 150 m representative reaches in each channel for 5 years during the early successional stage of the new stream-wetland complex (1st–6th years post-restoration) and prior to beaver colonization. Our macroinvertebrate monitoring program involved habitat-specific, quantitative estimates of invertebrate density and biomass. We placed special emphasis on insects from the Ephemeroptera, Plecoptera, and Trichoptera (EPT) orders because (1) EPT taxa are sensitive to a variety of stressors and thus are widely used in biological monitoring programs for use attainment (Barbour et al., 1999), and (2) we were curious how the regional pool of EPT taxa would assemble in lotic habitats of the newly restored stream-wetland complex.

## Methods

### Study sites

**Fig. 1:**
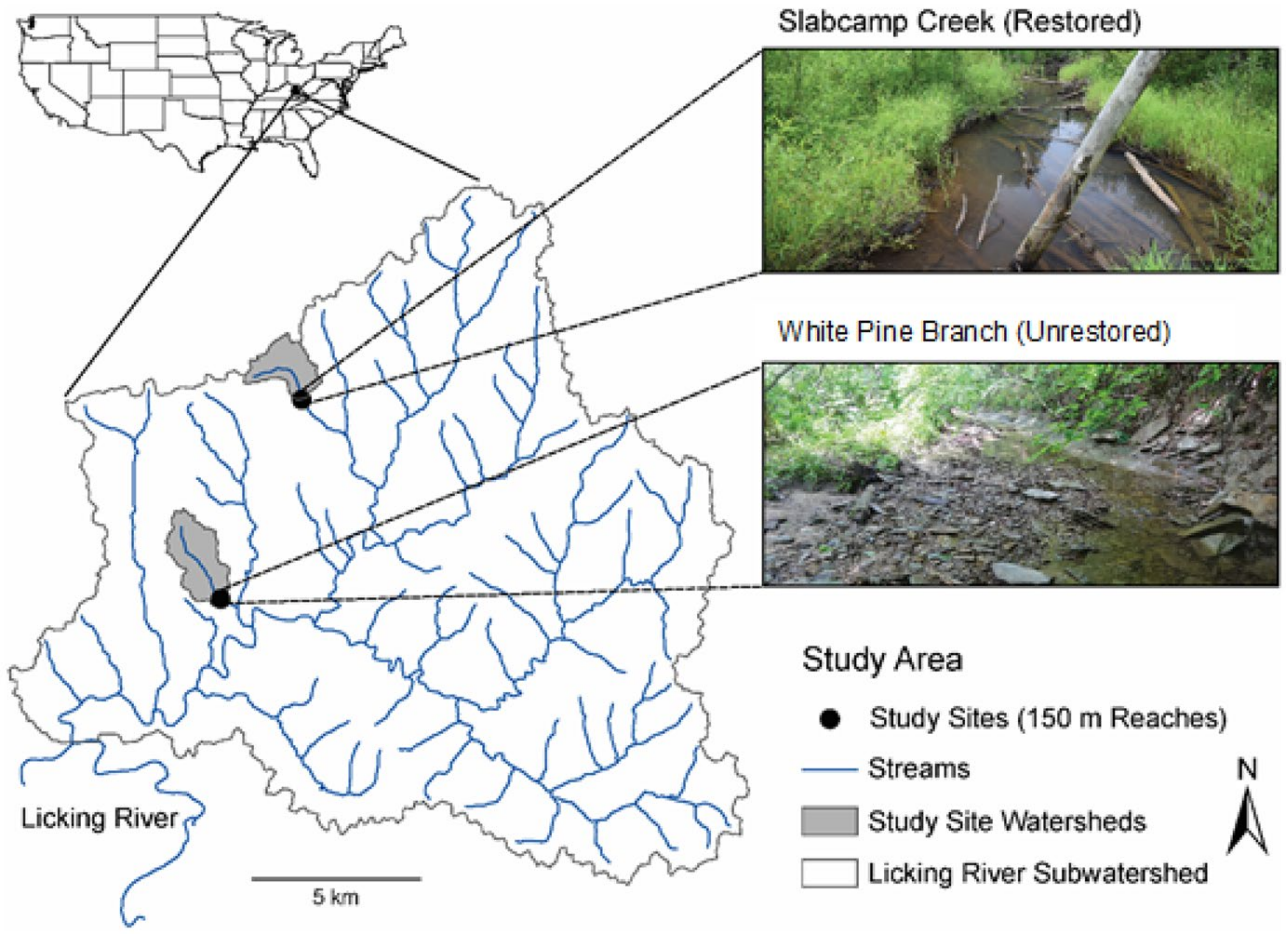
Location of study sites within the Licking River Drainage, KY, USA

This study occurred in two, first order tributaries of the North Fork Licking River watershed in the Western Allegheny ecoregion (Omernik, 1987) (Fig. 1). The streams drain small (< 3 km^2^), predominately forested watersheds within the Daniel Boone National Forest (Table 1). The watersheds have been relatively undisturbed for the past 80 years, have good water quality, and have no evidence of historical mining.

**Table 1:** Watershed and physical habitat characteristics from restored and unrestored sites observed within the monitoring period

	Restored	Unrestored
Watershed		
Area (km^2^)	2.3	2.4
% forest	80	99
% developed (low intensity)	4	1
% pasture/hay	14	
Wetted reach habitat		
Mean wetted width (m)	2.4	2.2
Mean wetted area (m^2^)	357	327
Mean % pool	57	34
Mean % riffle	14	41
Large woody debris (frequency)	100	30
Bed particles		
D_50_ (mm)	41	59
% sand	6	15
% gravel	74	38
% cobble	19	42
% boulder	0	5

% land cover was determined from 2019 National Land Cover Data (Dewitz, 2021)

**Fig. 2:**
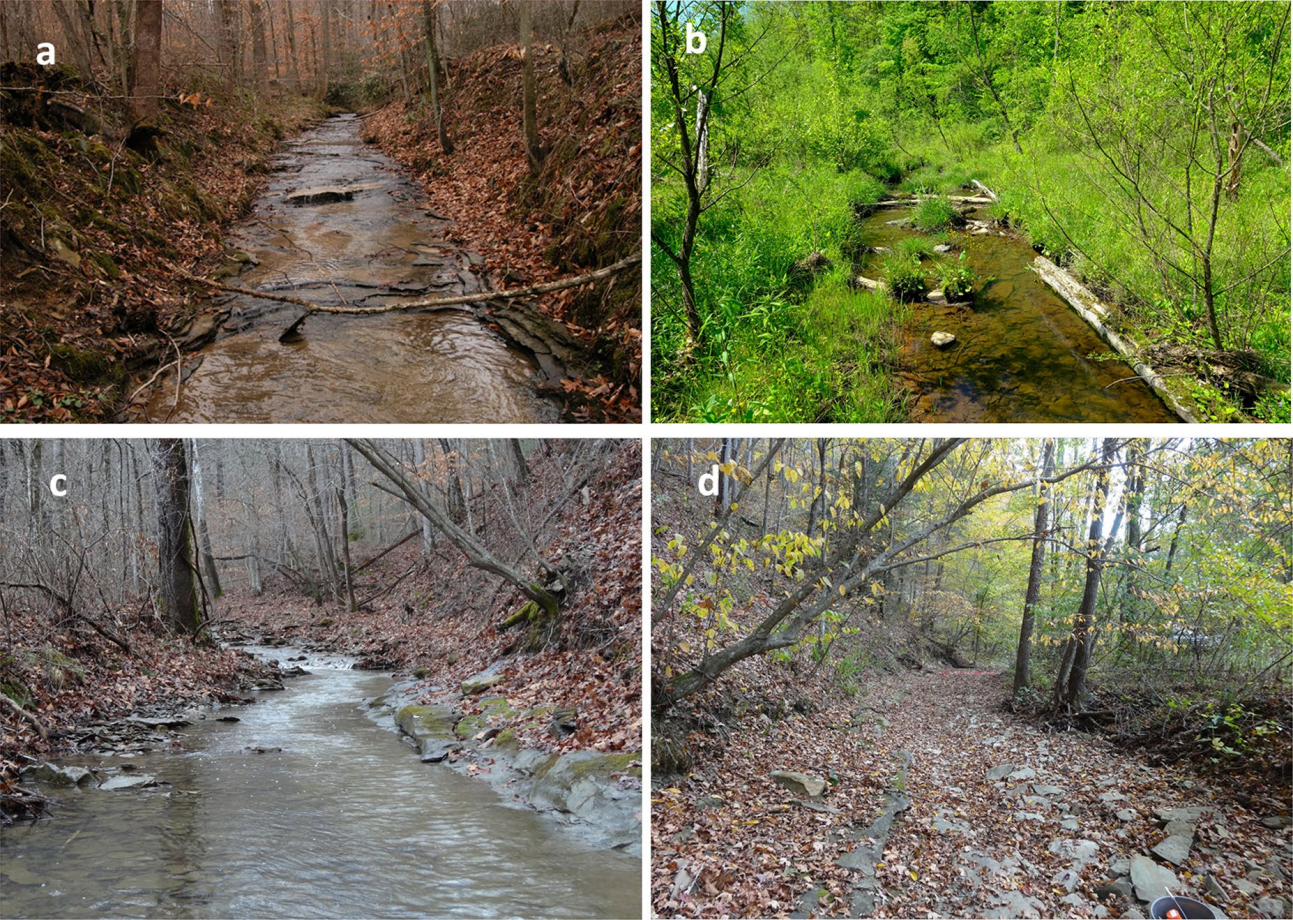
Photographic evidence of habitat conditions at the restored site relative to single-threaded headwater reaches in the Daniel Boone National Forest, USA: **a** restored site before restoration, winter; **b** restored site post-restoration, late summer; **c** unrestored site, winter; **d** unrestored site, late summer

The region experienced extensive logging and farming in the early to mid-1900s. Evidence of hydrologic impairment from historical land use exists throughout the national forest and includes the presence of historic farm fields in valleys, straight single-threaded channels relocated to the edge of the valleys, vertical eroding stream banks, channel beds dominated by exposed weathering shale bedrock and valley lag deposits, intermittent annual flow patterns, and incising stream networks from frequent headcutting. Test pits from the historical floodplains of the study sites revealed post-settlement alluvium that varied in thickness from less than a few centimeters to about 1.5 m. Disconnections from the floodplain and aquifer resulted in prolonged and extensive channel drying during late summer and fall. Channel velocities were sufficiently high to cause frequent mobilization of bed sediments during flood flows. Ultimately, these alterations resulted in single-threaded channels with homogenized, frequently disturbed habitat and limited refuge for the aquatic organisms (Fig. 2a, c, d).

The goal of the restoration project at Slabcamp Creek was to recreate the hydrologic functions that would have been present in the pre-settlement streams. Specifically, the restoration aimed to build a channel connected to the historical valley aquifer within the groundwater aquifer to increase the duration of baseflow, build a wide, low floodplain that would be frequently inundated during flooding, and reduce velocities on the channel during flood events. This restoration was achieved by removing the majority of the post-settlement alluvium, which was placed along the hillside. A wide (12–18 m) inset floodplain was created with a small channel with low banks (< 0.3 m) within that floodplain. The elevation of the baseflow channel was based on the elevation of the gravel layer identified during the geotechnical examination of the floodplain. The post-settlement alluvium removed to create the floodplain was placed against the hillsides to form ponds that collect overland flow from adjacent hillslopes and are connected to the groundwater during periods of the year when the water table is high. Tree trunks, root wads, and large limbs removed during the exaction process were installed within the channel to establish and maintain grade control and to provide additional habitat for fish and macroinvertebrates. The total linear distance of the restoration was 3.8 km and construction was largely complete by December 2011. A variety of new aquatic habitats was available for macroinvertebrate colonization upon completion of the restoration, including a main channel (with riffles, runs, and pools), side channels, woody debris dams, and depressional ponds in the floodplain.

### Monitoring design

Post-restoration hydrologic and biological monitoring was conducted in a 150-m stream reach of Slabcamp Creek (restored site; Fig. 2b). For a comparison, we also monitored hydrologic functions and macroinvertebrate assemblages from a 150-m reach of White Pine Branch (unrestored site; Fig. 2c, d), which served as the unrestored control site.

Flow duration and bed mobility were measured in each reach over 5 years, beginning in the 1st (July 2012) through 6th-year post-restoration (July 2017). The water stage was continuously measured at a 5-min interval in each reach using pressure transducers. Channel pressure transducer locations, floodplain, and channel topography were surveyed using a total station to determine periods of drying and flooding. Changes to the channel and floodplain topography were monitored during repeat visits to the site throughout the period of biological monitoring.

The mobility of coarse sediments in excess of 2 mm was assessed through the installation of traps which were vertically oriented and open at the channel surface. Traps were paired with a continuously recording seismic impact sensor to provide an improved measurement of the movement of coarse sediments during floods (Park, 2013; Rickenmann & McArdell, 2007). Sediment disturbance was classified into two categories: trace and significant movement. Trace movement was defined as seismic particle impacts numbering less than one hundred and sediment traps that were less than full during collection visits. The significant movement included particle impacts in excess of one hundred and full sediment traps.

Quantitative benthic samples were collected from a 150-m reach of each study site on eight separate occasions during the 3rd (18 December 2013; 9 March 2014; 4 August 2014), 4th (12 December 2014; 24 March 2015; 1 May 2015; 14 August 2015), and 5th (16 March 2016) years post-restoration. During each sampling event, five replicate samples were taken from randomly selected riffles and from five random pool channel units (sensu Church, 1992) with a Hess sampler (0.09 m^2^, 243-µm mesh). Only riffles, pools, and stretches of bedrock with the shallow flow during high-baseflows were available for sampling at the unrestored site. We monitored macroinvertebrate assemblages only from riffles and pools because they were the only comparable habitats between the study sites. Contents from the Hess were rinsed into plastic bags and preserved with 95% ethanol and transported to the laboratory for analysis. Our habitat-specific sampling program resulted in a total of 130 benthic samples throughout the study (restored riffles *n* = 30, unrestored riffles *n* = 30, restored pools *n* = 35, unrestored pools *n* = 35).

Each benthic sample was elutriated and rinsed through two stacked sieves (1 mm and 250 µm mesh sizes) in the laboratory. Invertebrates were sorted under a dissecting microscope from both fractions, but time-prohibitive samples from the 250 µm fraction were split with a volumetric sample splitter. Invertebrates were identified to the lowest practical taxonomic level, enumerated, and measured to the nearest 0.5 mm in order to estimate standing stocks of invertebrate biomass from published length-mass regressions (Benke et al., 1999).

The wetted habitat available for macroinvertebrates was determined during benthic sampling events. We measured the riffle and pool area, as well as wetted channel width at 11 equally spaced transects. During summer 2009, the number of pieces of large wood within 1 m^2^ of each transect was counted in order to estimate the frequency of large woody debris in each reach. Surface sediment textures were determined from modified Wolman pebble counts (Wolman, 1954).

### Statistical analysis

We used a mixed-effects model to compare the macroinvertebrate assemblage richness, density, and biomass between the restored and unrestored sites. Time (fall 2013, summer 2014, fall 2014, winter 2014, late-spring 2015, summer 2015, and winter 2016), stream (unrestored vs. restored), and channel unit (CU; riffle vs. pool) were the categorical fixed effect variables. The individual samples per channel unit were treated as the random effect variable. The fixed effect variables influence the mean of our response variables (Zuur et al., 2009). Our random effects influence the variance, which were the five samples nested in either the riffle or pool channel units. Treating the nested samples as a random effect allows us to account for the spatial pseudoreplication in our study design (Zuur et al., 2009). We analyzed riffle and pool samples separately because riffles were not sampled in summer 2014 (i.e., no water and subsequently no CU to sample); our mixed-effects models were simplified to address these missing data. Second, a pair-wise least square mean comparison using the Tukey’s post-hoc analysis was used to determine significant differences between the restored and unrestored riffles and the restored and unrestored pools. Density and biomass were log-transformed (log_10_ [x + 1]) to reduce heteroscedasticity. These analyses were completed in R (R Core Team, 2017) using the *lme* function (nlme package; Pinheiro et al., 2013) and the pair-wise comparisons using the *emmeans* function (emmeans package; Lenth, 2021; Searle et al., 1980).

Non-metric multidimensional scaling (NMDS) with the Bray–Curtis similarity coefficient was used to explore potential differences in assemblage structure in four a-priori channel unit groups (restored riffles, unrestored riffles, restored pools, unrestored pools). Replicates from each channel unit on each sampling date were combined and considered a sample unit, and taxa with low frequency (< 4%) were not included in the analysis. The final ordination matrix included 28 samples (rows) × 100 taxa (columns). First, we explored similarity in community structure and performed the ordination by calculating the Bray–Curtis similarity coefficients from the log _10_ (X + 1) invertebrate abundance data. Following the ordination analysis, we tested the null hypotheses of no differences in assemblage structure (absolute) among groups with a non-parametric multi-response permutation procedure (MRPP), which provides a *p*-value and an *A*-value. *A*-values from MRPP comparisons are a measure of effect size and greater values indicate stronger differences in assemblages. Finally, an indicator species analysis (ISA; Dufrêne & Legendre, 1997) was performed on the absolute abundance data to identify representative taxa from our a priori groups. The significance of each taxon’s indicator value (IV) was tested with Monte Carlo tests with 1000 permutations. The NMDS, MRPP, and ISA analyses were conducted using PC-ORD Version 6.0 (McCune & Mefford, 2011).

## Results

### Hydrologic functions

**Fig. 3:**
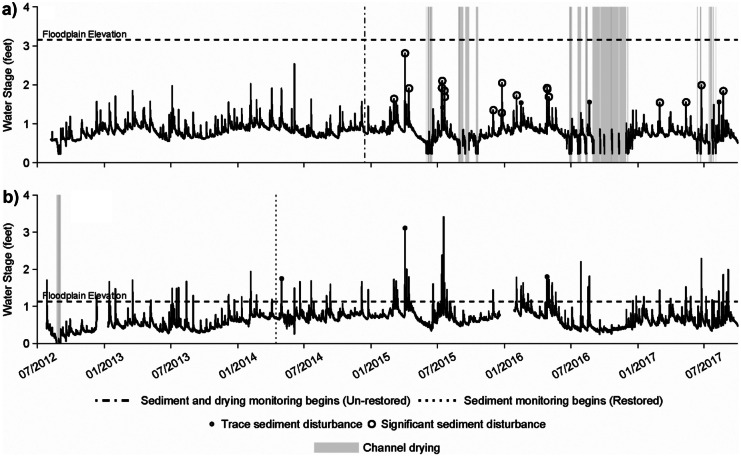
Hydrologic monitoring results from the **a** unrestored site and **b** restored site during the 1st through 6th years post-restoration

The unrestored site dried repeatedly during the late spring through late fall, with the earliest drying occurring in May of 2015 and the latest occurring in December of 2016. Drying events ranged from a few hours to weeks (Fig. 3a). Periods of drying were frequently interrupted by short periods of flow driven by thunderstorms. Drying was widespread throughout the unrestored site, with the channel frequently showing a loss of flow and residual water in pools within a few hours of drying onset. The unrestored site reach did not flood out of channel banks during the period of hydrologic monitoring. The restored site flowed continuously throughout the period of monitoring with the exception of a very brief period immediately following construction in the summer of 2012. The restored site flooded frequently during each year of monitoring with flooding durations ranging from hours to days (Fig. 3b).

Coarse sediment texture was similar between the two sites, with a higher median diameter for the unrestored site due to the greater presence of cobble and boulder material from exposed valley lag deposits (Table 1). In the unrestored site, sediment was frequently disturbed, most often during the late winter through summer (Fig. 3a). Both trace and significant sediment movement were recorded at the unrestored site, with the transport of cobble and boulder material observed during field visits. Only trace sediment movement was observed at the restored site (Fig. 3b). Throughout the duration of monitoring, sediment traps in the restored site were never full, a strong indication that channel velocities were successfully reduced.

### Macroinvertebrate assemblages

**Fig. 4:**
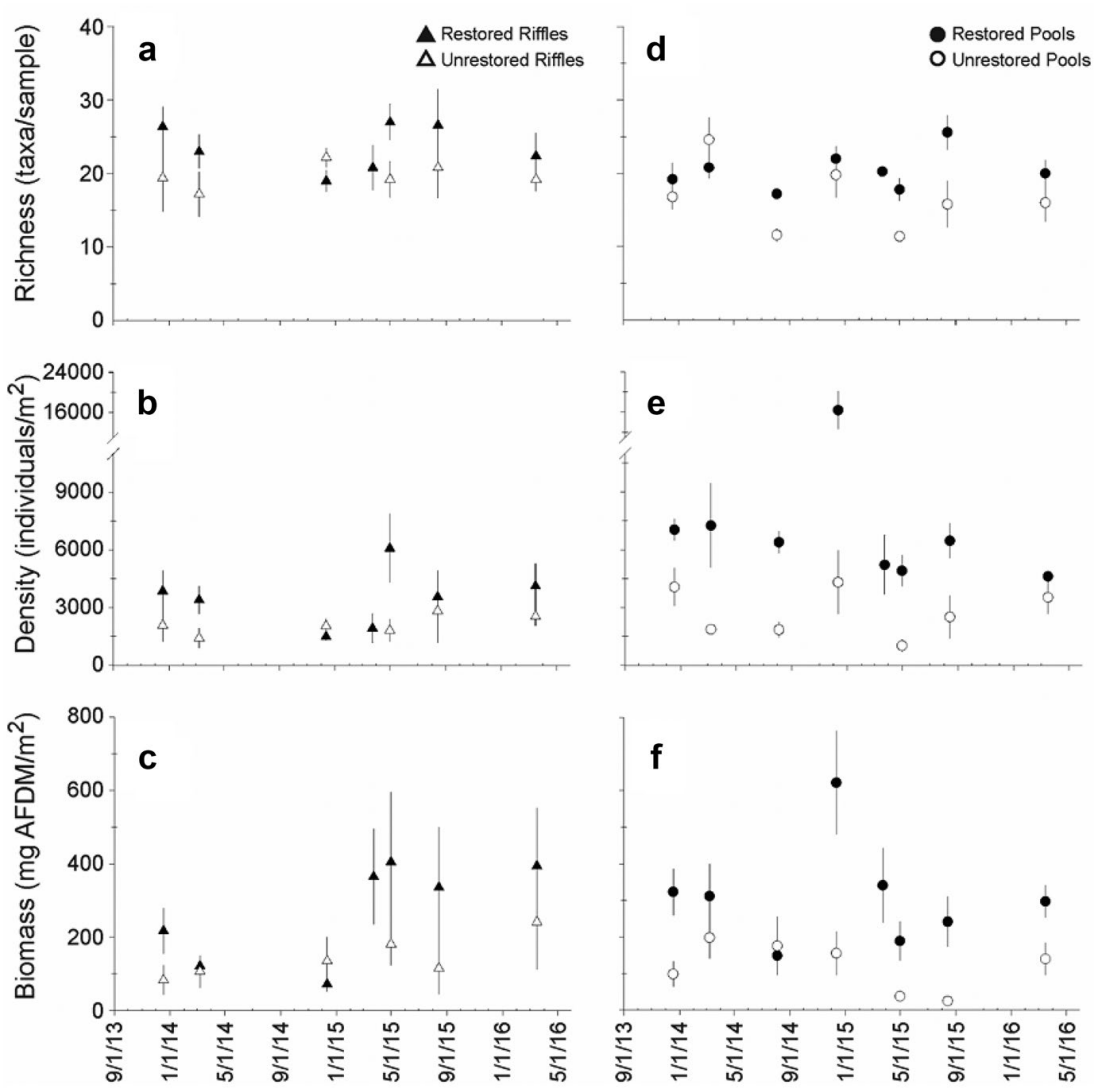
Mean (± SE) invertebrate **a**, **d** richness; **b, e** density; and **c, f** biomass between riffles (*n* = 5) and pools (*n* = 5) of the restored and unrestored sites over the multi-year monitoring period

We identified 112 total taxa and 58 EPT taxa throughout the entire study (Table S1). A total of 98 taxa and 50 EPT taxa were collected from the restored site; 99 taxa and 52 EPT taxa were collected from the unrestored site (Table S1). Seventy-seven percent of the taxa (86 total taxa) in our collection were collected from both sites; 13 taxa (including four Plecoptera taxa and two Trichoptera taxa) were unique to the restored site, and 14 taxa (including two Ephemeroptera, one Plecoptera, and five Trichoptera taxa) were collected exclusively from the unrestored site (Table S1). Results from mixed models indicated significantly greater taxa richness from riffles and pools of the restored site compared to riffles and pools of the unrestored site (Table 2; Fig. 4a, d), but we did not detect a significant difference in EPT taxa richness between the sites (Table 2).

**Table 2:** Mixed model results for total taxa richness, EPT richness, and total invertebrate density and biomass between channel units of the restored and unrestored sites

	Riffles			Pools		
	*df*	F	*p*	*df*	F	*p*
*Total richness*						
Intercept	1,44	**249.584**	**< 0.001**	1,52	**1056.025**	**< 0.001**
Time	5,44	0.562	0.729	6,52	**3.812**	**0.003**
Stream	1,44	**6.997**	**0.011**	1,52	**14.462**	**< 0.001**
Time X stream	5,44	1.179	0.335	6,52	1.810	0.115
*EPT richness*						
Intercept	1,44	**377.297**	**< 0.001**	1,52	**447.713**	**< 0.001**
Time	5,44	0.450	0.811	6,52	**3.491**	**0.006**
Stream	1,44	1.243	0.271	1,52	3.730	0.059
Time X stream	5,44	1.348	0.262	6,52	1.322	0.264
*Total density*						
Intercept	1,44	**249.584**	**< 0.001**	1,52	**557.393**	**< 0.001**
Time	5,44	0.561	0.654	6,52	**2.945**	**0.015**
Stream	1,44	**6.997**	**0.011**	1,52	**49.836**	**< 0.001**
Time X stream	5,44	1.179	0.335	6,52	0.891	0.509
*Total biomass*						
Intercept	1,44	40.711	< 0.001	1,52	**125.934**	**< 0.001**
Time	5,44	1.277	0.291	6,52	**3.883**	**0.003**
Stream	1,44	**4.131**	**0.048**	1,52	**44.082**	**< 0.001**
Time X stream	5,44	0.751	0.590	6,52	2.219	0.056

Bold values indicate significant differences

We collected 32,309 total individuals (9816 EPT individuals) and 1574 mg AFDM total invertebrate biomass (873 mg AFDM EPT biomass) from the restored site and 13,594 individuals (5330 EPT individuals) and 725 mg AFDM total invertebrate biomass (397 EPT biomass) from the unrestored site (Table S1). Collections from both reaches were numerically dominated by Chironomids (42% restored site; 37% unrestored site) and oligochaete worms (13% restored site, 10% unrestored site). Results from mixed models also indicated significantly greater total invertebrate density and biomass from the riffles and pools of the restored site (Table 2), but the effect was greater from restored pools (Fig. 4b, c, e, f), which supported three to four times the mean invertebrate density and biomass than unrestored pools (Fig. 4e, f). When the mixed model analysis was repeated with only the density and biomass of EPT taxa, we still detected greater density and biomass from restored pools but no significant difference between restored and unrestored riffles (Table 3).

**Table 3:** Mixed model results for density and biomass of aggregate and individual Ephemeroptera, Plecoptera, and Trichoptera taxa density and biomass between channel units of the restored and unrestored sites

	Riffles						Pools				
		Density		Biomass				Density		Biomass	
	*df*	F	*p*	F	*p*	*df*		F	*p*	F	*p*
*EPT*											
Intercept	1,44	**156.484**	**< 0.001**	**32.123**	**< 0.001**	1,52		**297.323**	**< 0.001**	**85.653**	**< 0.001**
Time	5,44	0.887	0.498	**2.747**	**0.030**	6,52		1.381	0.240	**7.368**	**< 0.001**
Stream	1,44	1.710	0.198	2.905	0.095	1,52		**31.516**	**< 0.001**	**53.257**	**< 0.001**
Time X stream	5,44	1.260	0.298	0.933	0.469	6,52		1.461	0.210	0.673	0.672
*Ephemeroptera*											
Intercept	1,44	**82.626**	**< 0.001**	**20.243**	**< 0.001**	1,52		**175.954**	**< 0.001**	**58.670**	**< 0.001**
Time	5,44	1.491	0.212	**2.706**	**0.032**	6,52		**2.446**	**0.037**	**4.561**	**0.001**
Stream	1,44	1.669	0.203	3.017	0.089	1,52		**55.107**	**< 0.001**	**67.410**	**< 0.001**
Time X stream	5,44	1.131	0.358	1.376	0.252	6,52		0.996	0.438	0.900	0.502
*Plecoptera*											
Intercept	1,44	**113.682**	**< 0.001**	**15.202**	**< 0.001**	1,52		**47.408**	**< 0.001**	**10.302**	**0.002**
Time	5,44	**5.046**	**0.001**	**5.220**	**0.001**	6,52		**6.024**	**0.001**	**5.778**	**< 0.001**
Stream	1,44	1.897	0.175	0.543	0.465	1,52		**5.866**	**0.019**	**7.070**	**0.010**
Time X stream	5,44	**3.993**	**0.005**	0.775	0.573	6,52		2.066	0.073	1.255	0.294
*Trichoptera*											
Intercept	1,44	**28.316**	**< 0.001**	8.190	0.006	1,52		**13.550**	**0.001**	**4.375**	**0.041**
Time	5,44	1.283	0.288	1.175	0.337	6,52		**10.516**	**< 0.001**	**5.899**	**< 0.001**
Stream	1,44	**6.557**	**0.014**	1.626	0.209	1,52		3.876	0.054	2.814	0.100
Time X stream	5,44	0.570	0.723	1.028	0.413	6,52		2.116	0.067	0.402	0.874

Bold values indicate significant differences

**Fig. 5:**
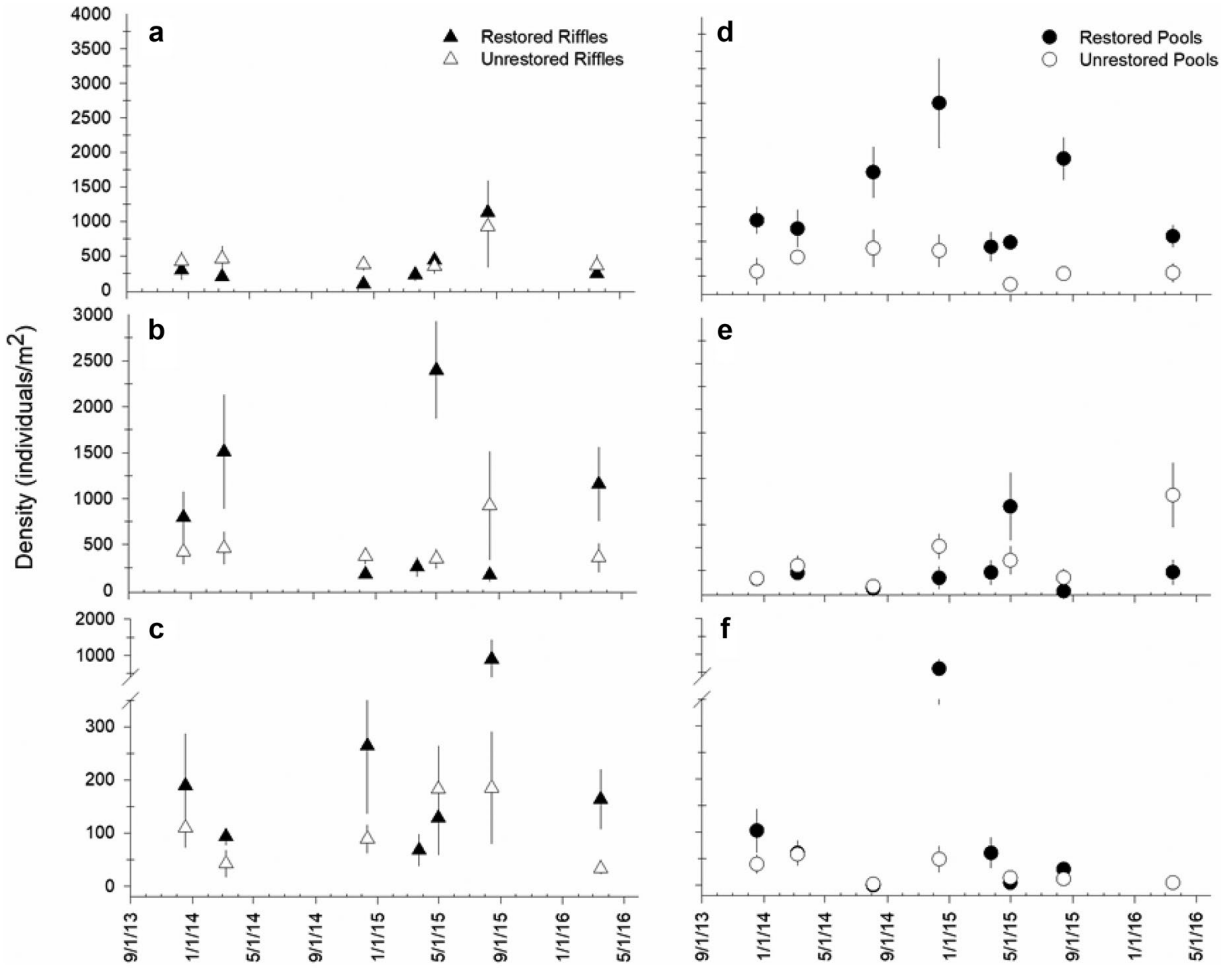
Mean (± SE) density of the insect orders **a**,** d** Ephemeroptera; **b**,** e** Plecoptera; and **c**,** f** Trichoptera between riffles (*n* = 5) and pools (*n* = 5) of the restored and unrestored sites over the multi-year monitoring period

**Fig. 6:**
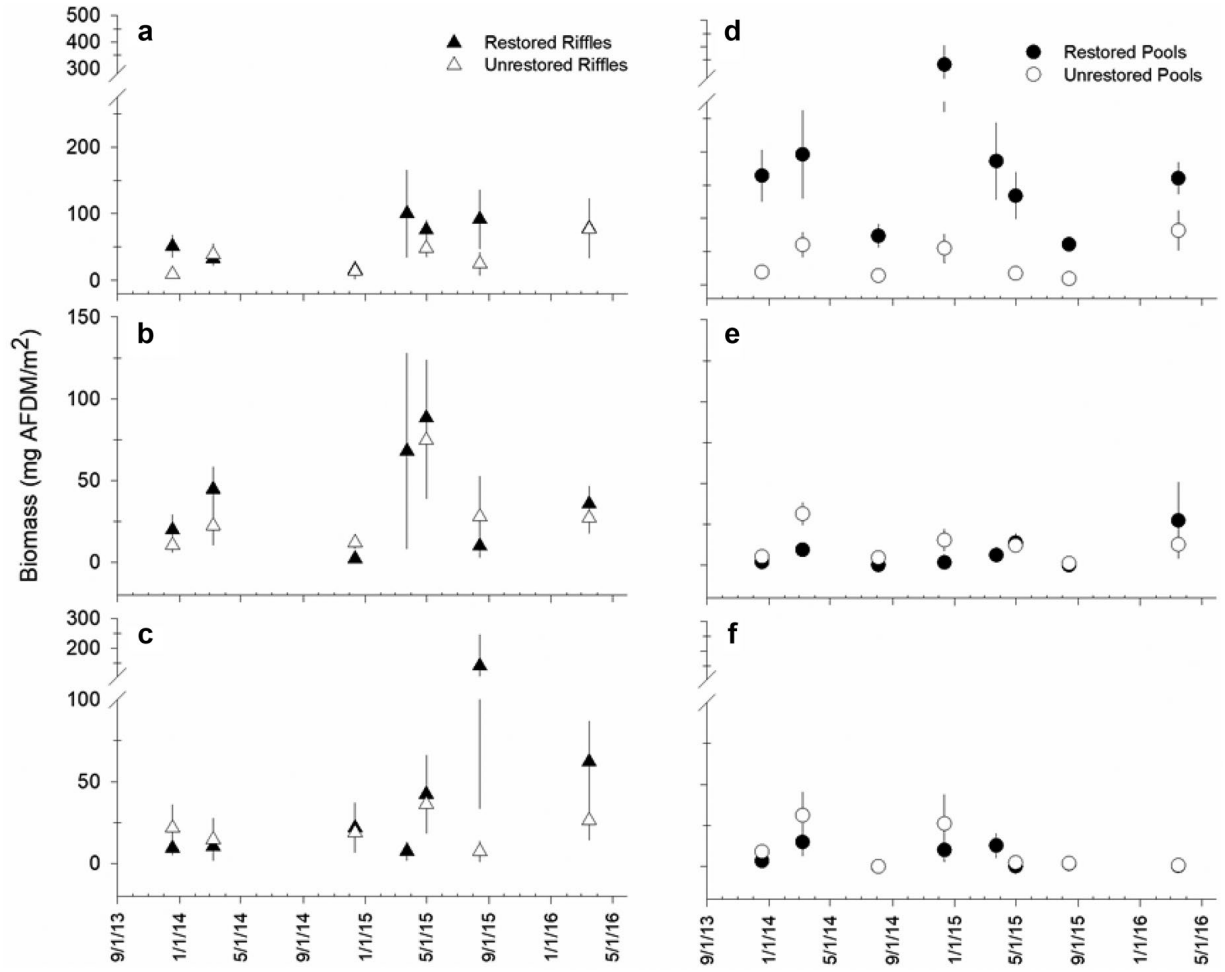
Mean (± SE) biomass of the insect orders **a**,** d** Ephemeroptera; **b**,** e** Plecoptera; and **c**,** f** Trichoptera between riffles (*n* = 5) and pools (*n* = 5) of the restored and unrestored sites over the multi-year monitoring period

Analysis of individual EPT orders revealed the difference in aggregate EPT density between restored and unrestored sites was driven by consistently greater mayfly density and biomass from the restored pools (Table 3; Figs. 5 and 6). The mayfly assemblage from restored pools was numerically dominated by *Ephemera*, *Caenis*, and *Stenacron* (Table S1). Total invertebrate biomass from restored pools was consistently dominated by *Ephemera*, which contributed 31–73% of mayfly biomass and 8–44% to total invertebrate biomass throughout the entire study.

**Fig. 7:**
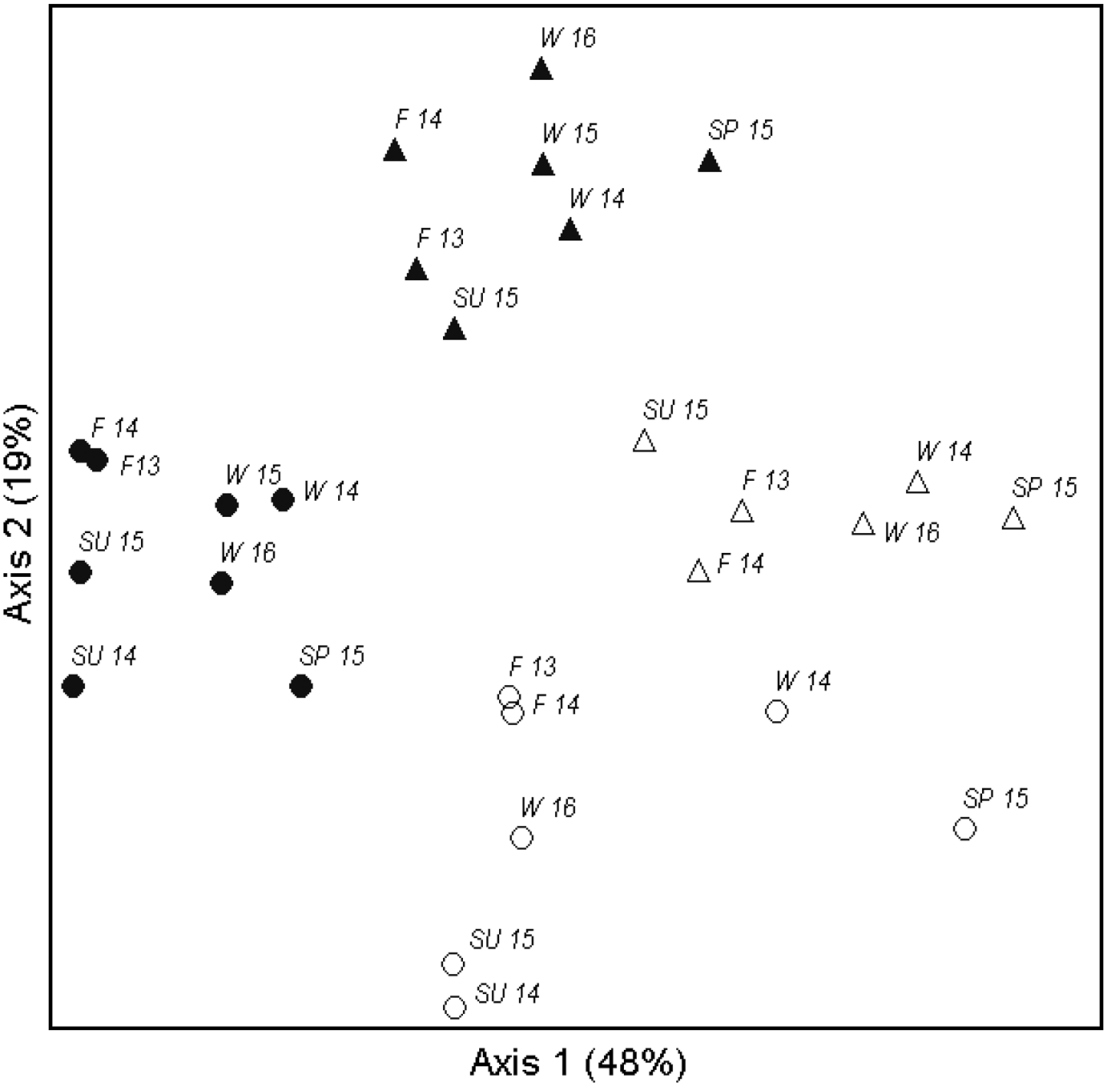
Nonparametric multidimensional scaling ordination analysis of macroinvertebrate abundance from restored riffles (black triangle), unrestored riffles (white triangle), restored pools (black circle), and unrestored pools (white circle). Labels indicate season (SU, summer; F, fall; W, winter; SP, spring) and year (2013, 13; 2014, 14; 2015, 15; 2016, 16). Stress = 10.5. MRPP results for habitat comparisons within and between sites: restored riffles vs. restored pools *A* = 0.15, *p* < 0.0001, unrestored riffles vs. unrestored pools *A* = 0.04, *p* = 0.0207; restored riffles vs. unrestored riffles *A* = 0.07, *p* = 0.0005, restored pools vs. unrestored pools *A* = 0.15, *p* = 0.0001

NMDS produced a 3-dimensional solution with low stress (10.5). Axis 1 (48%), Axis 2 (19%) and Axis 3 (15%) collectively explained 82% of the variation in the data. Visual examination of the ordination plots revealed differences in assemblages between sites, greater dispersion (variation) in the assemblage from the unrestored site, and more distinct differences between riffles and pools of the restored site than the unrestored site (Fig. 7). MRPP comparisons supported our interpretation and yielded stronger within site differences from the restored site (restored riffles vs. restored pools *A* = 0.15, *p* < 0.0001) compared to within site differences from the unrestored site (unrestored riffles vs. unrestored pools *A* = 0.04, *p* = 0.0207). MRPP comparisons also revealed greater differences between pools of the sites (restored pools vs. unrestored pools *A* = 0.15, *p* = 0.0001) rather than riffles (restored riffles vs. unrestored riffles *A* = 0.07, *p* = 0.0005). Axis 1 of the ordination (Fig. 7) explained most of the variation in assemblages (48%) and was driven by a gradient of collector-gatherers associated with slower currents and fine substrates (*Ephemera r* = − 0.83, *Stenacron* = − 0.82, *Caenis r* = − 0.78, ostracods *r* = − 0.79, copepods *r* = − 0.71, chironomids *r* = − 0.66,) to rheophilic taxa typical of riffles in headwater reference reaches in the region (*Epeorus r* = 0.77, *Cinygmula r* = 0.69, *Crangonyx r* = 0.69, *Haploperla r* = 0.68, *Diplectrona* = 0.66) (Pond, 2010, 2012).

Results from the ISA produced 35 significant (*p* < 0.05) indicator taxa (Table 4). Nearly half of the indicator taxa (16 taxa), some of which included the highest IVs, were from restored pools. Several mayflies — including taxa that burrow (*Ephemera*) or sprawl (*Caenis*) — were near-perfect (> 90% IndVal) indicators of restored pools. Other strong indicators (> 60% IndVal) of restored pools included the collector-gathering ostracods and *Dubiraphia* (a beetle). The mayfly *Eurylophella* was the only relatively weak indicator (48% IndVal) of unrestored pools. The top indicators (> 60% IndVal) of restored riffles were collector-filterers (i.e., *Isonychia*, *Cheumatopsyche*, *Chimarra*, and *Prosimulium*), predaceous flies (i.e., *Hemerodromia* and *Antocha*), and one scraping beetle (*Stenelmis*). The top indicators of unrestored riffles included collector-filtering caddisflies (i.e., *Diplectrona*, *Wormaldia*), a predaceous caddisfly (*Rhyacophila*), and the collector-gathering and scraping mayflies, *Diphetor* and *Epeorus*.

**Table 4:** Indicator values from channel units of the restored and unrestored sites

		Restored		Unrestored		*p*
		Riffle	Pool	Riffle	Pool	
Oligochaeta		22	53	7	18	0.004
Mollusca	*Ferrissia*	12	54	2	0	0.020
	Sphaeriidae	16	55	1	9	0.027
Crustacea	Cambaridae	6	56	1	9	0.004
	Copepoda	1	70	1	23	0.020
	*Crangonyx*	1	1	51	23	0.026
	Ostrocoda	10	82	0	3	0.001
Ephemeroptera	*Caenis*	5	94	0	0	0.001
	*Diphetor*	7	5	64	18	0.003
	*Ephemera*	3	92	1	4	0.001
	*Ephemerella*	51	0	3	0	0.026
	*Eurylophella*	5	20	7	48	0.013
	*Isonychia*	62	1	3	0	0.024
	*Epeorus*	17	0	60	3	0.006
	*Stenacron*	0	97	0	1	0.001
	*Stenonema*	1	87	0	10	0.001
Odonata	*Argia*	20	62	2	0	0.001
	*Gomphus*	0	42	0	2	0.022
Megaloptera	*Corydalus*	54	1	0	0	0.007
	*Sialis*	0	56	2	0	0.008
Coleoptera	*Dubiraphia*^A,L^	0	84	0	0	0.001
	*Optioservus*^A,L^	11	1	48	15	0.032
	*Stenelmis*^A,L^	71	8	2	5	0.002
Trichoptera	*Rhyacophila*	6	2	65	2	0.001
	*Cheumatopsyche*	65	5	3	2	0.010
	*Diplectrona*	2	0	85	5	0.001
	*Chimarra*	96	0	1	0	0.001
	*Dolophilodes*	1	0	57	1	0.010
	*Wormaldia*	2	0	60	1	0.017
Diptera	*Antocha*	71	0	0	0	0.002
	*Bezzia/Palpomyia*	11	66	6	17	0.002
	Chironomidae	16	56	10	18	0.001
	*Hemerodromia*	74	9	2	1	0.001
	*Hexatoma*	6	0	49	27	0.023
	*Prosimulium*	68	1	15	1	0.008

Only taxa with significant *p*-values following Monte Carlo tests are shown

## Discussion

The goal of the Slabcamp Creek restoration was to restore hydrologic functions, including reconnecting the channel to its historic valley aquifer, constructing a small channel that would frequently inundate its floodplain, and to reduce flood velocities in the channel and floodplain. Evidence from hydrologic monitoring during the post-restoration years shows that the restoration was successful at restoring these functions. First, the restored connection to the aquifer extended the flow duration through the critical flow period (summer and fall) when many headwaters in our region experience periods of channel drying, including the unrestored site. Second, flood velocities were reduced, which led to a reduction of coarse sediment movement and increased the retention of organic matter in the channel.

We detected differences in macroinvertebrate assemblages between the restored and unrestored sites, but due to our inability to sample prior to the restoration, we cannot be certain the differences were due solely to the restoration. However, empirical evidence from hydrologic monitoring coupled with our years of experience with macroinvertebrate assemblages from forested, single-threaded headwaters in the region and wetlands throughout the southeastern USA, we are confident that the magnitude of difference between the assemblages from the study sites was largely a result of environmental change following restoration rather than inherent differences in assemblages before the restoration and throughout the monitoring period.

Quantitative collections from pools and aggregate functional metrics (density, biomass) revealed the greatest differences in macroinvertebrate assemblages between the restored and unrestored sites. Our estimates of macroinvertebrate biomass indicate potential food available for insectivorous fish per square meter of stream bottom at time points throughout the study. Habitat-weighted estimates are particularly useful here because they allow for comparison among reaches with vastly different habitats. A quick extrapolation of our sample-scale biomass estimates (mg AFDM/m^2^) to the total amount of wetted area covered by riffles and pools during the monitoring period indicates macroinvertebrate biomass from riffles and pools of the restored reach (34–147 g AFDM) was 2–4 × greater than the amounts from unrestored reach (14–34 g AFDM). These rough estimates are especially conservative for the restored reach because we observed aquatic insects on habitats and substrates that were not included in our sampling design. Preliminary sampling and consistent observations throughout the monitoring period indicated few, if any, macroinvertebrates (and other aquatic life, e.g., mosses) on bedrock sheets from the unrestored site.

Consistently greater standing stocks of macroinvertebrate biomass from the restored site throughout this study — especially during summer and fall when aquatic habitat from single-threaded headwaters dry and disconnect — have implications for the valley food webs. Indeed, fish surveys from both study sites during the 3rd and 4th years post-restoration yielded greater frequency, abundance (with a greater percentage of the assemblage comprised of insectivorous individuals), and richness of fish from the restored site (Mike Compton, Office of Kentucky Nature Preserves, personal communication). Additionally, aerial stages of aquatic insects are known to subsidize riparian food webs (Schindler & Smits, 2016). Assuming our instream estimates translated to greater emergence production from the restored reach, it would be reasonable to hypothesize dietary benefits to terrestrial consumers (e.g., birds, bats).

Removal of some second-growth stands of woody vegetation was a necessary part of restoring hydrologic functions to the headwater valley. Forest canopy adjacent to single-threaded headwaters may mediate temperatures and provide allochthonous inputs that support detrital-based food webs (Meyer & Wallace, 2001). On the other hand, legacy effects in the form of simplified habitat with reduced capacity to retain forest litter inputs (Bilby & Likens, 1980; Muotka & Laasonen, 2002; Webster et al., 1994) can alter the biological structure and function of channelized streams (Lepori et al., 2005; Pilotto et al., 2018) regardless of canopy cover from reforested riparian zones (Harding et al., 1998).

Because the aquatic biota from forested headwaters may be limited by habitat and bottom-up factors (i.e., light, nutrients), the physical and biological changes brought about by canopy removal, the new flow regime, and habitat (structure and stability) probably had complex and perhaps confounding effects on the assemblage from the restored site. Without a rigorous study design that incorporates basal food resources, nutrients, and physical–chemical factors (e.g., temperature, dissolved oxygen), we cannot effectively partition the relative influence of hydrologic improvement and canopy removal on the assemblage from the restored site. Some attributes of the restored assemblages (greater densities and biomass) may indicate subsidy effects and an altered temperature regime as a result of canopy removal. For example, indicator taxa from riffles of the unrestored site might suggest unfavorable conditions for rheophilic, oxygen-loving taxa associated with cool water (*Epeorus*, *Diphetor*, *Diplectrona*, *Rhyacophila*, *Dolophilodes*, and *Optioservus*), while taxa with a wider tolerance to temperature were associated with the restored site.

On the other hand, attributes of the restored assemblages reflect habitat improvements following the restoration (e.g., perennial water, greater bed stability, and retention of organic matter). Our direct measures of sediment analysis and movement provide evidence of homogenized habitat and biota that has been widely reported from channelized streams (Lau et al., 2006; Maul et al., 2004; Moyle, 1976; Negishi et al., 2002). For example, riffles and pools of the restored site supported distinct subassemblages that were not as apparent from the unrestored site. This distinction was evident from the unrestored site during high-baseflow periods (winter 2014, spring 2015) when unrestored pools shifted along NMDS Axis 1 and became more similar to riffles — especially during the most significant bed movement event during spring 2015. Furthermore, the subassemblage from restored pools included taxa with feeding modes and life history attributes that are well suited for slower current and fine sediments that are typical of pool habitats in lotic systems. Burrowing mayflies (*Ephemera*) were particularly important because they drove the differences in EPT abundance and biomass and were among the strongest indicators of restored pools. Burrowing mayflies excavate burrows in finer sediments where they exist and collect organic matter, which aligns with greater channel habitat stability, sediment, and organic matter retention in the restored site. They are also among the longest-lived mayflies (up to 2 years) and develop slowly over multiple seasons (McCafferty, 1975), which also aligns with longer flow duration and more stable hydrologic conditions from the restored site.

Regardless of the causal mechanisms, we regard the structure and function of the macroinvertebrate assemblages from the restored site during the early post-restoration years as indicative of a positive ecological lift from the restored valley, especially considering the potential impacts to higher consumers (Schindler & Smits, 2016). Flitcroft et al. (2022) reached a conclusion similar to ours after their review of post-restoration monitoring studies from Stage 0 channels in the Pacific Northwest. More specifically, secondary production estimates indicated a greater capacity to support higher trophic levels during the first year following the restoration of the South Fork McKenzie River (Flitcroft et al., 2022). As expected, beaver recolonized the restored valley shortly after this study (7th year post-restoration), and although we are not certain of the trajectory, we expect aquatic habitats in the restored valley will continue to support subassemblages of macroinvertebrates that will adjust according to the dynamic nature of self-sustaining, beaver-controlled systems. We continue to perform critical monitoring necessary to understand ecological responses to restored hydrologic functions through the second stage of the new valley’s succession.

### Implications for biological assessments from restored stream-wetland complexes

Quantitative biomonitoring studies such as ours are not practical for all stream-wetland complex restorations. Rapid, cost-effective tools that serve as proxies for desired outcomes would help guide restoration practices (Kollmann et al., 2016; Meyer et al., 2015). Rubin et al. (2017) concluded that improper selection of biological endpoints — particularly biological indices for water quality — is contributing to the lack of documented ecological improvement following many stream restorations. Along the same lines, our habitat-specific findings raise concern regarding the utility of widely used reach-scale macroinvertebrate sampling methodologies (i.e., Rapid Bioassessment Protocols; Barbour et al., 1999;) to assess improved hydrologic functions following the restoration of stream-wetland complexes.

Several lines of evidence from this case study suggest that if we had relied solely on RBP methods without supporting evidence from hydrologic monitoring, then we likely would have concluded no difference in assemblages between the study sites and possibly even a degraded assemblage from the restored site. First, reach-scale assessment and monitoring designs report the relative abundance (% composition) and richness of macroinvertebrate taxa from a composite of samples collected from multiple riffles or other habitats along a reach. Taken together, the important mayfly assemblages driving the functional differences between sites would not have been detected if the riffle and pool samples were combined, and we did not consider the absolute abundance of taxa from these habitats. We agree with recent calls from others to consider habitat-specific abundance and biomass of macroinvertebrates in post-restoration assessments (Dolph et al., 2015; Herbst et al., 2018). Second, in contrast to functional metrics, richness-based metrics did not indicate strong differences in assemblage structure between the restored and unrestored site; we did not detect a difference in EPT richness between sites and only slightly greater taxa richness from the restored site. Furthermore, total taxa and EPT richness values from both reaches were within the range of the values expected from single-threaded, reference reaches in our region (Pond et al., 2003). This result should not be surprising because the unrestored site had attributes of reaches that represent best-attainable conditions (i.e., no history of mining, forested watersheds, good water quality) that also experienced historical land use disturbance.

The findings from restored pools in our case study indicate that attributes of macroinvertebrate assemblages should serve as robust indicators of hydrologic improvements following the restoration of stream-wetland complexes. It should not be surprising that several mayfly taxa were among the most important indicators of restored and unrestored habitat because species within the order tolerate a wide range of depths, current, speeds, and substrates (Brittain & Saltveit, 1989). Mayfly assemblages respond to a variety of stressors (Jacobus et al., 2019 and references therein), and they serve as important food resources for a variety of consumers (Grant, 2001). Therefore, the abundance, richness, and life history traits of mayfly assemblages will be given special consideration as we continue biological assessments of restored stream-wetland complexes.

## Conclusions

Our findings from invertebrate and hydrologic monitoring in this case study provide insight into how the pool of macroinvertebrate taxa from forested watersheds will assemble in newly restored stream-wetland complexes in our region. We also demonstrate how the assemblage differed from a contemporary incised channel. We found a unique assemblage of mayflies and a positive ecological lift (based on abundance and biomass) from pools of the restored site. Results from our post-restoration biological monitoring demonstrate the importance of habitat-specific sampling and suggest metrics based on functional (density and biomass) rather than structural (taxonomic richness) attributes of invertebrate assemblages may be robust indicators of improved hydrologic functions following restoration. Collective results from various geographic regions and watershed contexts might reveal similar assemblage attributes that can be developed into scientifically sound, robust bioindicators of hydrologic improvements following the restoration of stream-wetland complexes. We encourage restoration practitioners, regulatory agencies, and the scientific community to think carefully about expected biological endpoints from restored stream-wetland complexes before applying RBP methods that were not designed to assess ecological responses to restored hydrologic functions.

## Supplementary Information

Below is the link to the electronic supplementary material.

Supplementary file1 (DOCX 40 kb)

## Data Availability

All invertebrate data generated or analyzed during this study are included in Figs. 4–7, Tables 1–4, and the supplementary table. The hydrologic data in Fig. 3 are available from the corresponding author on reasonable request.

## References

[CR1] Barbour, M. T., Gerritsen, J., Snyder, B. D., & Stribling, J. B. (1999). Rapid bioassessment protocols for use in streams and wadeable rivers: Periphyton, benthic macroinvertebrates, and fish. Office of Water, U.S. Environmental Protection Agency, Washington DC.

[CR2] Benke AC, Huryn AD, Smock LA, Wallace JB (1999). Length-mass relationships for freshwater macroinvertebrates in North America with particular reference to the southeastern United States. Journal of the North American Benthological Society.

[CR3] Bilby RE, Likens GE (1980). Importance of organic debris dams in the structure and function of stream ecosystems. Ecology.

[CR4] Brittain JE, Saltveit SJ (1989). A review of the effect of river regulation on mayflies (Ephemeroptera). Regulated Rivers: Research & Management.

[CR5] Burchsted D, Daniels M, Thorson R, Vokoun J (2010). The river discontinuum: Applying beaver modifications to baseline conditions for restoration of forested headwaters. BioScience.

[CR6] Buss DF, Carlisle DM, Chon TS, Culp J, Harding JS, Keizer-Vlek HE, Robinson WA, Strachan S, Thirion C, Hughes RM (2015). Stream biomonitoring using macroinvertebrates around the globe: A comparison of large-scale programs. Environmental Monitoring and Assessment.

[CR7] Chapman MG (1998). Improving sampling designs for measuring restoration in aquatic habitats. Journal of Aquatic Ecosystem Stress and Recovery.

[CR8] Church M, Carlow P, Petts GE (1992). Channel morphology and typology. The Rivers Handbook.

[CR9] Cluer B, Thorne C (2014). A stream evolution model integrating habitat and ecosystem benefits. River Research and Applications.

[CR10] Collins BD, Montgomery DR, Sheikh AJ, Montgomery DR, Bolton S, Booth DB, Wall L (2003). Reconstructing the historic riverine landscape of the Puget Lowland. Restoration of Puget Sound Rivers.

[CR11] Dewitz, J. (2021). National Land Cover Database (NLCD) 2019 Products [Data set]. U.S. Geological Survey. 10.5066/P9KZCM54

[CR12] Dolph CL, Eggert SL, Magner J, Ferrington LC, Vondracek B (2015). Reach-scale stream restoration in agricultural streams of southern Minnesota alters structural and functional responses of macroinvertebrates. Freshwater Science.

[CR13] Dufrêne M, Legendre P (1997). Species assemblages and indicator species: the need for a flexible asymmetrical approach. Ecological Monographs.

[CR14] Elliott SJ, Wilf P, Walter RC, Merritts DJ (2013). Subfossil leaves reveal a new upland hardwood component of the pre-European Piedmont landscape, Lancaster County, Pennsylvania. PLoS ONE.

[CR15] Flitcroft RL, Brignon WR, Staab B, Bellmore JR, Burnett J, Burns P, Cluer B, Giannico G, Helstab JM, Jennings J, Mayes C, Mazzacano C, Mork L, Meyer K, Munyon J, Penaluna BE, Powers P, Scott DN, Wondzell SM (2022). Rehabilitating valley floors to a stage 0 condition: A synthesis of opening outcomes. Frontiers in Environmental Science.

[CR16] Foster D, Swanson F, Aber J, Burke I, Brokaw N, Tilman D, Knapp A (2003). The importance of land-use legacies to ecology and conservation. BioScience.

[CR17] Goerman D, Krauss R, Jayakumar D, Bernstein M (2013). Wetland and stream mitigation: Application of a resource condition assessment protocol in the Pennsylvania Marcellus Shale. Environmental Geosciences.

[CR18] Grant PM, Dominguez E (2001). Mayflies as food. Trends in research in Ephemeroptera and Plecoptera.

[CR19] Harding JS, Benfield EF, Bolstad PV, Helfman GS, Jones EBD (1998). Stream biodiversity: The ghost of land use past. Proceedings of the National Academy of Sciences.

[CR20] Harman, W., Starr, R., Carter, M., Tweedy, K., Clemmons, M., Suggs, K., & Miller, C. (2012). *A function-based framework for stream assessment and restoration projects*. EPA 843-K-12-006. US Environmental Protection Agency, Washington, D.C.

[CR21] Harwood K, Brown AG (1993). Fluvial processes in a forested anastomosing river: Flood partitioning and changing flow patterns. Earth Surface Processes and Landforms.

[CR22] Herbst DB, Cooper SD, Medhurst RB, Wiseman SW, Hunsaker CT (2018). A comparison of the taxonomic and trait structure of macroinvertebrate communities between the riffles and pools of montane headwater streams. Hydrobiologia.

[CR23] Huryn AD, Wallace JB (2000). Life history and production of stream insects. Annual Review of Entomology.

[CR24] Jacobus LM, Macadam CR, Sartori M (2019). Mayflies (Ephemeroptera) and their contributions to ecosystem services. Insects.

[CR25] Kaushal SS, McDowell WH, Wollheim WM (2014). Tracking evolution of urban biogeochemical cycles: Past, present, and future. Biogeochemistry.

[CR26] Kollmann J, Meyer ST, Bateman R, Conradi T, Gossner MM, de Souza Mendonça M, Fernandes GW, Hermann J, Koch C, Müller SC, Oki Y, Overbeck GE, Paterno GB, Rosenfield MF, Toma TSP, Weisser WW (2016). Integrating ecosystem functions into restoration ecology—Recent advances and future directions. Restoration Ecology.

[CR27] Kramer N, Wohl EE, Harry DL (2012). Using ground penetrating radar to ‘unearth’ buried beaver dams. Geology.

[CR28] Lau JK, Lauer TE, Weinman ML (2006). Impacts of channelization on stream habitats and associated fish assemblages in east central Indiana. The American Midland Naturalist.

[CR29] Lenth, R. V. (2021). Emmeans: Estimated marginal means, aka least-squares means. R package version 1.6.3. R Project for Statistical Computing, Vienna, Austria.

[CR30] Lepori F, Palm D, Malmqvist B (2005). Effects of stream restoration on ecosystem functioning: Detritus retentiveness and decomposition. Journal of Applied Ecology.

[CR31] Maul JD, Farris JL, Milam CD, Cooper CM, Testa SIII, Feldman DL (2004). The influence of stream habitat and water quality on macroinvertebrate communities in degraded streams of northwest Mississippi. Hydrobiologia.

[CR32] McCafferty WP (1975). The burrowing mayflies (Ephemeroptera: Ephemeroidea) of the United States. Transactions of the American Entomological Society.

[CR33] McCune, B., & Mefford, M. J. (2011). *PC-ORD. Multivariate analysis of ecological data* version 6. MjM Software, Gleneden Beach, Oregon.

[CR34] Meyer JL, Wallace JB, Press MC, Huntly NJ, Levin S (2001). Lost linkages and lotic ecology: Rediscovering small streams. Ecology: Achievement and Challenge.

[CR35] Meyer ST, Koch C, Weisser WW (2015). Towards a standardized rapid ecosystem function assessment (REFA). Trends in Ecology & Evolution.

[CR36] Moyle PB (1976). Some effects of channelization on the fishes and invertebrates of Rush Creek, Modoc County, California. California Fish and Game.

[CR37] Muotka T, Laasonen P (2002). Ecosystem recovery in restored headwater streams: The role of enhanced leaf retention. Journal of Applied Ecology.

[CR38] Naiman RJ, Johnston CA, Kelley JC (1988). Alteration of North American streams by beaver. BioScience.

[CR39] Negishi JN, Inoue M, Nunokawa M (2002). Effects of channelisation on stream habitat in relation to a spate and flow refugia for macroinvertebrates in northern Japan. Freshwater Biology.

[CR40] Omernik JM (1987). Ecoregions of the conterminous United States. Annals of the Association of American Geographers.

[CR41] Park, J. W. (2013). *Piezoelectric bedload impact sensor (PBIS) for particle size distribution*. PhD Dissertation, University of Louisville, Louisville.

[CR42] Parola AC, Hansen C (2011). Reestablishing groundwater and surface water connections in stream restoration. Sustain.

[CR43] Pilotto F, Nilsson C, Polvi LE, McKie BG (2018). First signs of macroinvertebrate recovery following enhanced restoration of boreal streams used for timber floating. Ecological Applications.

[CR44] Pinheiro, J., Bates, D., DebRoy S., & Sarkar, S. (2013). Package nlme: Linear and nonlinear mixed effect models. R package version 3.1–109. R Project for Statistical Computing, Vienna, Austria.

[CR45] Pond GJ (2010). Patterns of Ephemeroptera taxa loss in Appalachian headwater streams (Kentucky, USA). Hydrobiologia.

[CR46] Pond GJ (2012). Biodiversity loss in Appalachian headwater streams (Kentucky, USA): Plecoptera and Trichoptera communities. Hydrobiologia.

[CR47] Pond, G. J., Call, S. M., Brumley, J. F., & Compton, M. C. (2003). *The Kentucky macroinvertebrate bioassessment index: Derivation of regional narrative ratings for wadeable and headwater streams*. Kentucky Department for Environmental Protection, Division of Water, Frankfort, Kentucky.

[CR48] R Core Team. (2017). *R: A language and environment for statistical computing*. R Foundation for Statistical Computing, Vienna, Austria. Retrieved May 2019, from https://www.R-project.org/

[CR49] Resh VH, McElravy EP, Rosenberg DM, Resh VH (1993). Contemporary quantitative approaches to biomonitoring using benthic macroinvertebrates. Freshwater Biomonitoring and Benthic Macroinvertebrates.

[CR50] Rickenmann D, McArdell BW (2007). Continuous measurement of sediment transport in the Erlenbach stream using piezoelectric bedload impact sensors. Earth Surface Processes and Landforms.

[CR51] Rubin Z, Kondolf GM, Rios-Touma B (2017). Evaluating stream restoration projects: What do we learn from monitoring?. Water.

[CR52] Schindler DE, Smits AP (2016). Subsidies of aquatic resources in terrestrial ecosystems. Ecosystems.

[CR53] Searle SR, Speed FM, Milliken GA (1980). Population marginal means in the linear model: An alternative to least squares means. The American Statistician.

[CR54] Stoddard JL, Larsen DP, Hawkins CP, Johnson RK, Norris RH (2006). Setting expectations for the ecological condition of streams: The concept of reference condition. Ecological Applications.

[CR55] Wallace JB, Webster JR (1996). The role of macroinvertebrates in stream ecosystem function. Annual Review of Entomology.

[CR56] Walter RC, Merritts DJ (2008). Natural streams and the legacy of water-powered mills. Science.

[CR57] Webster JR, Covich AP, Tank JL, Crockett TV (1994). Retention of coarse organic particles in streams in the southern Appalachian Mountains. Journal of the North American Benthological Society.

[CR58] Wohl E (2004). Disconnected rivers, linking rivers to landscapes.

[CR59] Wohl E, Beckman ND (2014). Leaky rivers: Implications of the loss of longitudinal fluvial disconnectivity in headwater streams. Geomorphology.

[CR60] Wolman MG (1954). A method of sampling coarse river-bed material. Transactions American Geophysical Union.

[CR61] Zuur AF, Ieno EN, Walker N, Saveliev AA, Smith GM (2009). Mixed effects models and extensions in ecology with R.

