# Supplementary material for: Macroinvertebrate assemblages from a stream-wetland complex: a case study with implications for assessing restored hydrologic functions
Source: Environ Monit Assess. 2023 Feb 13;195(3):394. doi: 10.1007/s10661-023-10983-7 (PMC9925594; doi:10.1007/s10661-023-10983-7)
Supplement: Supplementary file 1 — Supplementary file1 (DOCX 40 kb) [file 10661_2023_10983_MOESM1_ESM.docx]

Macroinvertebrate assemblages from a stream-wetland complex: a case study with implications for assessing restored hydrologic functions

**Authors and Addresses**

Amy Braccia^1^, Jamie Lau^2^, Jesse Robinson^3^, Michael Croasdaile^3^, Jeong Park^3^, Art Parola^3^

Corresponding author: Amy Braccia, [amy.braccia@eku.edu](mailto:amy.braccia@eku.edu); ORCID 0000-0001-7830-0992

^1^ Department of Biological Sciences, Eastern Kentucky University, Richmond, Kentucky U.S.A. 40475

^2^ Biology Department, Radford Univeristy, Radford, Virginia, U.S.A 24142

^3^Stream Institute, J.B. Speed School of Engineering, University of Louisville, Louisville, Kentucky, U.S.A. 40292

Table S1.

Taxa sum abundance and biomass from channels of the restored and un-restored reaches. Values are sums from samples collected throughout the multi-year monitoring period. *indicates rare taxa that were not included in NMDS analysis.

|  |  | RESTORED | | | |  | UNRESTORED | | | |
| --- | --- | --- | --- | --- | --- | --- | --- | --- | --- | --- |
|  |  | Riffle | | Pool | |  | Riffle | | Pool | |
| ORDER | FINALID | Abd | Bio | Abd | Bio |  | Abd | Bio | Abd | Bio |
| Non-Insecta | Cambaridae | 5 | − | 15 | 0.00 |  | 1 | 0.00 | 5 | 0.00 |
| Non-Insecta | Copepoda | 20 | − | 1284 | 0.00 |  | 15 | 0.00 | 438 | 0.00 |
| Non-Insecta | Crangonyx sp. | 8 | 0.14 | 8 | 0.01 |  | 125 | 14.22 | 71 | 5.12 |
| Non-Insecta | Ferrissia sp. | 9 | 0.12 | 37 | 1.22 |  | 2 | 0.03 | 1 | 0.01 |
| Non-Insecta | Gastropoda | 3 | 0.31 | 16 | 0.53 |  |  | 0.00 | 1 | 0.03 |
| Non-Insecta | Hydracarina | 76 | − | 26 | 0.00 |  | 17 |  | 13 | 0.00 |
| Non-Insecta | Lirceus sp. | 22 | 3.19 | 6 | 0.11 |  | 52 | 25.62 | 65 | 35.33 |
| Non-Insecta | Nematoda | 53 | − | 126 | 0.00 |  | 20 | 0.00 | 127 | 0.00 |
| Non-Insecta | Oligochaeta | 1072 | 11.49 | 3198 | 47.74 |  | 346 | 3.56 | 1043 | 7.29 |
| Non-Insecta | Ostrocoda | 24 | − | 225 | 0.00 |  | 2 | 0.00 | 9 | 0.00 |
| Non-Insecta | Planariidae |  |  | 4 | 0.02 |  | 1 | 0.01 | 1 | 0.01 |
| Non-Insecta | Sphaeriidae | 27 | 1.01 | 123 | 3.54 |  | 4 | 0.12 | 31 | 0.49 |
| Ephemeroptera | Acentrella sp. | 41 | 1.10 | 1 | 0.01 |  | 60 | 0.62 | 4 | 0.03 |
| Ephemeroptera | Acerpenna sp. | 136 | 3.96 | 173 | 3.13 |  | 28 | 0.50 | 2 | 0.06 |
| Ephemeroptera | Ameletus sp. | 14 | 1.44 | 2 | 0.35 |  | 18 | 1.63 | 192 | 24.50 |
| Ephemeroptera | Baetidae unk. | 58 | 0.44 | 84 | 0.68 |  | 119 | 0.95 | 9 | 0.07 |
| Ephemeroptera | Baetis sp. | 46 | 1.85 | 6 | 0.18 |  | 75 | 7.23 | 6 | 0.53 |
| Ephemeroptera | Caenis sp. | 56 | 1.04 | 1302 | 32.51 |  | 5 | 0.16 | 5 | 0.02 |
| Ephemeroptera | Centroptilum sp. |  |  | 3 | 0.12 |  |  |  | 1 | 0.09 |
| Ephemeroptera | Cinygmula sp. | 18 | 5.73 | 1 | 0.19 |  | 180 | 17.64 | 121 | 10.31 |
| Ephemeroptera | Diphetor sp. | 30 | 0.86 | 41 | 1.02 |  | 210 | 11.23 | 68 | 2.54 |
| Ephemeroptera | Drunella sp. | 7 | 1.95 |  |  |  | 1 | 0.31 |  |  |
| Ephemeroptera | Epeorus sp. | 37 | 5.30 | 1 | 0.00 |  | 134 | 13.99 | 16 | 1.66 |
| Ephemeroptera | Ephemera sp. | 34 | 7.40 | 1320 | 268.85 |  | 13 | 4.03 | 55 | 11.76 |
| Ephemeroptera | Ephemerella sp. | 31 | 6.88 |  |  |  | 1 | 0.04 | 1 | 0.04 |
| Ephemeroptera | Ephemerellidae unk |  |  |  |  |  | 2 | 0.02 |  |  |
| Ephemeroptera | Eurylophella sp. | 14 | 1.00 | 54 | 5.18 |  | 25 | 0.58 | 119 | 6.78 |
| Ephemeroptera | Habrophlebia sp. |  |  |  |  |  |  |  | 3 | 0.08 |
| Ephemeroptera | Heptageniidae unk. | 23 | 0.53 | 60 | 1.10 |  | 119 | 1.29 | 97 | 1.11 |
| Ephemeroptera | Heterocleon sp. | 35 | 0.71 |  |  |  | 21 | 0.38 | 2 | 0.09 |
| Ephemeroptera | Isonychia sp. | 139 | 32.53 | 5 | 0.52 |  | 14 | 1.58 |  |  |
| Ephemeroptera | Leptophlebiidae unk | 4 | 0.02 |  |  |  | 2 | 0.01 | 6 | 0.02 |
| Ephemeroptera | Leucrocuta sp.* |  |  |  |  |  |  |  | 10 | 0.90 |
| Ephemeroptera | Maccaffertium sp. | 188 | 72.32 | 88 | 32.51 |  | 71 | 20.77 | 70 | 29.21 |
| Ephemeroptera | Paraleptophlebia sp. | 78 | 1.90 | 326 | 3.69 |  | 129 | 7.59 | 366 | 14.96 |
| Ephemeroptera | Plauditus sp. | 21 | 0.55 | 1 | 0.05 |  | 5 | 0.18 |  |  |
| Ephemeroptera | Procloeon sp. | 6 | 0.13 | 66 | 1.17 |  |  |  | 11 | 0.34 |
| Ephemeroptera | Stenacron sp. | 3 | 0.05 | 519 | 71.76 |  | 1 | 0.04 | 11 | 0.06 |
| Ephemeroptera | Stenonema sp. | 2 | 0.26 | 229 | 56.83 |  |  |  | 29 | 5.90 |
| Odonata | Argia sp. | 20 | 0.72 | 51 | 28.14 |  | 6 | 0.04 |  |  |
| Odonata | Boyeria sp. | 3 | 34.28 | 1 | 1.08 |  | 2 | 6.95 | 2 | 49.20 |
| Odonata | Calopteryx sp.* | 1 | 0.03 |  |  |  |  |  |  |  |
| Odonata | Cordulegaster sp. |  |  | 2 | 0.13 |  | 1 | 0.04 | 8 | 1.09 |
| Odonata | Gomphidae unk. | 14 | 0.07 | 2 | 0.07 |  | 4 | 0.02 |  |  |
| Odonata | Gomphus sp. |  |  | 6 | 7.75 |  |  |  | 1 | 0.01 |
| Odonata | Lanthus sp. | 4 | 0.23 |  |  |  | 2 | 0.61 | 4 | 1.62 |
| Odonata | Stylogomphus sp. | 5 | 2.94 | 23 | 82.91 |  |  |  | 16 | 24.99 |
| Plecoptera | Acroneuria sp. | 58 | 12.31 | 1 | 10.22 |  | 43 | 39.57 | 3 | 0.00 |
| Plecoptera | Allocapnia sp. | 798 | 7.52 | 152 | 2.37 |  | 459 | 6.05 | 458 | 4.44 |
| Plecoptera | Alloperla sp.* | 1 | 0.04 |  |  |  |  |  |  |  |
| Plecoptera | Amphinemura sp. | 335 | 10.32 | 24 | 0.10 |  | 58 | 1.66 | 10 | 0.06 |
| Plecoptera | Chloroperlidae unk. | 152 | 2.78 | 64 | 1.86 |  | 89 | 2.91 | 51 | 0.59 |
| Plecoptera | Diploperla sp. | 2 | 4.95 |  |  |  |  |  | 3 | 1.42 |
| Plecoptera | Eccoptura sp. | 1 | 0.09 |  |  |  | 1 | 0.09 | 2 | 1.84 |
| Plecoptera | Haploperla sp. | 6 | 0.26 | 1 | 0.12 |  | 134 | 11.58 | 237 | 13.27 |
| Plecoptera | Isoperla sp. | 298 | 14.05 | 240 | 3.11 |  | 4 | 0.25 | 3 | 0.31 |
| Plecoptera | Leuctra sp. | 623 | 6.02 | 200 | 1.36 |  | 508 | 3.95 | 255 | 3.99 |
| Plecoptera | Malirekus sp.* | 2 | 1.00 |  |  |  |  |  |  |  |
| Plecoptera | Nemouridae unk. | 25 | 0.13 | 18 | 0.11 |  | 11 | 0.04 | 8 | 0.05 |
| Plecoptera | Ostrocerca sp. | 4 | 0.72 | 4 | 0.65 |  |  |  |  |  |
| Plecoptera | Paracapnia sp. |  |  |  |  |  | 33 | 3.39 | 44 | 3.54 |
| Plecoptera | Peltoperla sp. | 30 | 4.03 |  |  |  | 1 | 0.02 |  |  |
| Plecoptera | Perlesta sp.* | 1 | 0.18 |  |  |  | 1 | 0.18 |  |  |
| Plecoptera | Plecoptera unk. | 103 | 2.27312 | 54 | 0.66162 |  | 45 | 1.34162 | 34 | 0.77312 |
| Plecoptera | Prostoia sp. | 65 | 10.57 | 4 | 0.58 |  | 2 | 0.17 | 1 | 0.18 |
| Plecoptera | Sweltsa sp. | 35 | 6.48 | 8 | 2.33 |  | 18 | 3.41 | 23 | 5.00 |
| Plecoptera | Taenionema sp. | 105 | 1.05 |  |  |  | 1 | 0.01 |  |  |
| Plecoptera | Taeniopteryx sp. | 11 | 1.25 |  |  |  |  |  |  |  |
| Megaloptera | Corydalus sp. | 16 | 24.56 | 1 | 0.32 |  |  |  |  |  |
| Megaloptera | Nigronia sp. | 2 | 1.49 |  |  |  |  |  |  |  |
| Megaloptera | Sialis sp. |  |  | 12 | 5.62 |  | 1 | 0.28 |  |  |
| Coleoptera | Dubiraphia sp. A |  |  | 1 | − |  |  |  |  |  |
| Coleoptera | Dubiraphia sp. L | 2 | 0.13 | 122 | 15.28 |  | 2 | 0.03 |  |  |
| Coleoptera | Ectopria sp. |  |  |  |  |  | 6 | 1.75 | 2 | 0.03 |
| Coleoptera | Helichus sp. | 16 | 1.05 |  |  |  |  |  | 2 | 0.00 |
| Coleoptera | Hydrophilidae L* |  |  |  |  |  |  |  | 1 | 0.00 |
| Coleoptera | Optioservus sp. A |  |  |  |  |  | 2 | − |  |  |
| Coleoptera | Optioservus sp. L | 19 | 0.82 | 3 | 0.49 |  | 48 | 2.63 | 22 | 1.27 |
| Coleoptera | Oulimnius sp. A | 2 | − |  |  |  | 8 | − |  |  |
| Coleoptera | Oulimnius sp. L | 6 | 0.36 | 2 | 0.06 |  | 2 | 0.06 |  |  |
| Coleoptera | Psephenus sp. | 21 | 5.93 | 56 | 6.97 |  | 7 | 1.91 | 50 | 1.73 |
| Coleoptera | Stenelmis sp. A | 9 | − |  |  |  | 1 | − |  |  |
| Coleoptera | Stenelmis sp. L | 63 | 4.25 | 20 | 1.44 |  | 3 | 0.12 | 16 | 0.15 |
| Diptera | Antocha sp. | 6 | 1.11 |  |  |  |  |  |  |  |
| Diptera | Atrichopogon sp. |  |  | 1 | 0.00 |  | 2 | 0.00 | 2 | 0.00 |
| Diptera | Cecidomyiidae | 4 | 0.06 | 10 | 0.51 |  | 5 | 0.06 | 5 | 0.02 |
| Diptera | Ceratopogonidae | 156 | 0.60 | 1180 | 6.31 |  | 85 | 0.64 | 279 | 1.64 |
| Diptera | Chironomidae | 2801 | 23.55 | 10634 | 139.29 |  | 1570 | 11.60 | 3466 | 22.58 |
| Diptera | Chrysops sp. |  |  | 2 | 1.59 |  |  |  | 4 | 0.02 |
| Diptera | Culicidae |  |  | 2 | 0.00 |  |  |  |  |  |
| Diptera | Dasyhelea sp. | 31 | 0.02 | 37 | 0.02 |  | 4 | 0.00 | 12 | 0.00 |
| Diptera | Dicranota sp. | 4 | 1.77 |  |  |  | 2 | 0.61 |  |  |
| Diptera | Dixa sp. |  |  |  |  |  | 2 | 0.00 |  |  |
| Diptera | Ephydridae* | 1 | 0.00 |  |  |  |  |  |  |  |
| Diptera | Hemerodromia sp. | 80 | 1.63 | 18 | 0.29 |  | 7 | 0.07 | 5 | 0.13 |
| Diptera | Hexatoma sp. | 3 | 0.75 | 1 | 0.07 |  | 19 | 5.41 | 17 | 11.56 |
| Diptera | Limnophila sp.* |  |  |  |  |  |  |  | 1 | 0.35 |
| Diptera | Metachela sp. | 2 | 0.10 | 7 | 0.25 |  | 3 | 0.32 | 16 | 2.65 |
| Diptera | Molophilus sp. | 1 | 0.39 |  |  |  |  |  | 2 | 1.86 |
| Diptera | Neoplasta sp.* |  |  |  |  |  | 7 | 0.77 |  |  |
| Diptera | Pericoma sp. |  |  | 5 | 0.05 |  |  |  |  |  |
| Diptera | Prosimulium sp. | 408 | 8.22 | 8 | 0.36 |  | 80 | 2.01 | 8 | 0.11 |
| Diptera | Pseudolimnophila sp. |  |  |  |  |  | 2 | 0.23 | 1 | 0.03 |
| Diptera | Sciaridae |  |  | 1 | 0.00 |  | 2 | 0.04 | 7 | 0.12 |
| Diptera | Simulium sp. | 165 | 3.75 | 7 | 0.33 |  | 22 | 0.95 | 2 | 0.03 |
| Diptera | Tabanidae | 1 | 0.28 | 1 | 0.15 |  | 1 | 0.03 | 2 | 0.47 |
| Diptera | Tipula sp. | 12 | 169.66 | 2 | 42.64 |  | 6 | 68.99 | 2 | 8.26 |
| Diptera | Tipulidae | 1 | 0.03 | 9 | 1.19 |  | 3 | 0.08 | 2 | 0.07 |
| Trichoptera | Anisocentropus sp. |  |  |  |  |  | 1 | 3.93 | 3 | 6.57 |
| Trichoptera | Cheumatopsyche sp. | 57 | 20.52 | 7 | 1.39 |  | 4 | 2.65 | 6 | 2.07 |
| Trichoptera | Chimarra sp. | 252 | 33.20 | 3 | 0.08 |  | 8 | 0.15 |  |  |
| Trichoptera | Diplectrona sp. | 11 | 7.78 | 1 | 0.03 |  | 103 | 30.46 | 9 | 4.59 |
| Trichoptera | Dolophilodes sp. | 1 | 0.51 |  |  |  | 10 | 4.15 | 1 | 0.03 |
| Trichoptera | Glossosoma sp. |  |  |  |  |  | 2 | 1.18 |  |  |
| Trichoptera | Hydropsyche sp. | 21 | 11.61 | 1 | 0.65 |  | 2 | 1.18 | 1 | 0.01 |
| Trichoptera | Hydropsychidae unk | 71 | 0.36 | 1 | 0.01 |  | 3 | 0.83 |  |  |
| Trichoptera | Hydroptila sp. | 42 | 25.95 | 38 | 1.13 |  |  |  |  |  |
| Trichoptera | Lepidostoma sp. |  |  |  |  |  | 7 | 1.64 | 8 | 1.82 |
| Trichoptera | Lype sp.* |  |  |  |  |  |  |  | 1 | 0.57 |
| Trichoptera | Neophylax sp. | 276 | 20.00 | 281 | 2.16 |  | 39 | 0.64 | 25 | 0.19 |
| Trichoptera | Oecetis sp.* |  |  | 3 | 0.01 |  |  |  |  |  |
| Trichoptera | Polycentropus sp. | 1 | 0.03 | 2 | 0.13 |  | 1 | 0.03 | 3 | 0.11 |
| Trichoptera | Psychomyia sp.* |  |  |  |  |  |  |  | 1 | 0.02 |
| Trichoptera | Pycnopsyche sp. | 1 | 0.12 | 7 | 3.68 |  | 1 | 1.29 | 12 | 12.57 |
| Trichoptera | Rhyacophila sp. | 5 | 1.61 | 2 | 4.00 |  | 14 | 3.75 | 2 | 1.97 |
| Trichoptera | Wormaldia sp. | 4 | 0.65 |  |  |  | 81 | 2.81 | 5 | 0.19 |
|  |  |  |  |  |  |  |  |  |  |  |
| Total |  | 9616 | 661.345 | 22693 | 913.098 |  | 5419 | 369.909 | 8175 | 355.531 |
